# MOF‐74 Nanofibers as an Advanced Porous Material for Air‐Bearing Technology

**DOI:** 10.1002/smll.202411108

**Published:** 2025-07-02

**Authors:** Jacopo Andreo, Xiangjiang Yu, Ekaterina Chernova, Stefan Wuttke

**Affiliations:** ^1^ BCMaterials Basque Center for Materials Applications and Nanostructures UPV/EHU Science Park Leioa Vizcaya 48940 Spain; ^2^ Academic Centre for Materials and Nanotechnology AGH University of Krakow al. Adama Mickiewicza 30 Kraków 30‐059 Poland

**Keywords:** air‐bearings, fibers, MOF‐74, nanofibers, porous composites, self‐assembly

## Abstract

Aerostatic bearings are an important technology that utilizes a thin film of pressurized air between bearing surfaces to enable frictionless movement. In this work, the use of MOF‐74 (Cu) fibers as an innovative material for porous restrictors in aerostatic bearing devices is proposed. MOF‐74 (Cu) fibers are synthesized using a newly developed, green, template‐free approach that produces ultra‐long and robust fibers with excellent mechanical properties. These fibers can be easily processed into free‐standing films and composites, offering a scalable and cost‐effective method for producing air‐bearing pucks. Tests under operational conditions for low‐pressure air bearings demonstrate the reliable structural and mechanical strength of the MOF‐74 (Cu) fibers, positioning them as a promising alternative to traditional bearing materials. This advancement not only sparks the development of air‐bearing technology, but also broadens the potential for MOFs in real‐world applications, paving the way for green and sustainable manufacturing solutions.

## Graphical abstract

This work introduces MOF‐74 (Cu) fibers as innovative porous restrictor materials, synthesized via a green, template‐free method yielding ultra‐long, robust fibers. Processable into free‐standing films, these fibers exhibit excellent mechanical strength under low‐pressure operational conditions, offering a scalable, sustainable alternative for advanced air‐bearing applications, a class of bearings relying on a pressurized air film to enable frictionless movement.

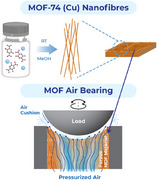

## 1 Introduction

Metal–organic frameworks (MOFs) are a class of porous materials composed of metal ions or clusters coordinated to organic ligands, forming highly ordered structures with tuneable pore sizes and surface areas.^[^ ^1^, ^2^ ^]^ Since their discovery, MOFs have been extensively explored for applications ranging from gas storage and separation to catalysis and drug delivery, owing to their high porosity, chemical tunability, and structural diversity.^[^ ^3^, ^4^ ^]^ In the last few years, the search for novel techniques to manufacture strong freestanding MOF films and monoliths has flourished, with the discovery of novel approaches or the adaptation of known techniques to these materials leading to the successful manufacturing and characterization of MOF and MOF/composite macroscopic devices.^[^ ^5^, ^6^ ^]^ Within this research frame, particle morphology has become even more important, as it is a key parameter for the techniques that rely on pre‐synthetized MOFs materials instead of in situ synthesis, often too slow or impractical for the production of macroscopic devices.^[^ ^7^ ^]^ In particular, nano‐ and micro‐scale morphologies of MOF particles, have attracted significant interest, as they can be precisely controlled to optimize the performance of these materials in various applications, achieving enhanced stability, adsorption kinetics, and mechanical behavior.^[^ ^8^, ^9^, ^10^ ^]^ However, the development of MOF fibers, specifically nanofibers, has proven to be more challenging compared to other morphologies, such as microcrystals or nanoparticles. MOF nanofibers, with their high aspect ratio and directional porosity, present unique advantages over more common MOF morphologies. Their elongated structure can enhance the diffusion of gases and guest molecules through the material, potentially improving adsorption and catalytic processes^[^ ^11^ ^]^ Despite these promising attributes, the fabrication of pure MOF nanofibers has remained difficult due to the need for templating agents or complex synthesis protocols. Most of the reported approaches rely on templated methods, which involve using pre‐existing fibrous substrates or frameworks to guide the formation of MOF fibers. These methods, while effective, are time‐consuming and limited in scalability, making them impractical for large scale production and thus hindering their applications. Consequently, a simple, scalable method to synthesize MOF fibers is highly desirable to unlock their full potential in industrial and technological applications.^[^ ^12^, ^13^ ^]^

To date, only a few examples of template‐free synthesis of 1D monocrystalline MOFs with high aspect ratios have been published.^[^ ^14^, ^15^ ^]^ In 2018, single‐crystal MOF‐74 (Co) nanotubes with lengths of up to 30 µm and diameters of ≈60 nm were synthesized via an amorphous MOF‐mediated recrystallization approach.^[^ ^16^ ^]^ This method involved first forming amorphous MOF‐74 (Co) nanoparticles at room temperature, followed by recrystallization into nanotubes in water at 175 °C. To the best of our knowledge, the only example of room‐temperature, template‐free synthesis of MOF nanofibers, is the synthesis of Cu₂(BTEC) in ethanol, which resulted in crystalline nanowires with lengths of 5 µm and diameters of 25 nm within 2 h.^[^ ^17^ ^]^ Interestingly, the characterization of this process revealed that alcohol molecules acted as structure‐directing templates, guiding the self‐assembly of the organic linkers and promoting nanowire formation. However, the structure of the obtained material was not fully characterized, as the combination of metal and linker used in the synthesis may lead to a plethora of possible topologies and crystal structures, none of which particularly resistant to moisture or mechanical stress.

In this work, we report a simple, room‐temperature synthesis of MOF‐74 (Cu) nanofibers that requires no templating agents or complex conditions. Produced in bulk, these nanofibers feature high aspect ratios and retain good crystallinity even after exposure to air and moisture. Their fibrous morphology emerges spontaneously during synthesis, with the nanofibers assembling into larger bundles that enhance flexibility in suspension. Despite the inherent brittleness of MOFs, the fibrous MOF‐74 (Cu) particles exhibit sufficient mechanical integrity to be directly cast into desired shapes, forming cohesive, free‐standing materials. Upon drying, the interwoven nanofibers create a mat‐like structure of precise and scalable thickness, with strong inter‐fiber bonding that prevents redispersion in solvents. These films exhibit brittle fracture under excessive stress but can be repaired by applying a nanofiber suspension to the fracture site. This opens new possibilities for integrating MOFs into advanced material applications.

One such application, which we explore as a proof‐of‐concept, is the use of MOF‐74 (Cu) nanofibers for the fabrication of aerostatic bearings. Air bearings represent an innovative class of contactless surface‐to‐surface bearings, where pressurized gas forms a thin cushion that separates the moving parts, eliminating friction, wear, and the need for lubrication. However, the widespread adoption of air bearings is currently limited by the high cost and complexity of manufacturing the porous materials required for the bearing surfaces. Traditional materials such as sintered ceramics and graphite have significant drawbacks: ceramics are prone to catastrophic failure due to brittleness, while graphite generates hazardous dust during fabrication.^[^ ^18^, ^19^, ^20^, ^21^, ^22^ ^]^

**Figure 1:**
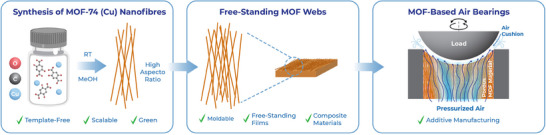
Overview and novelty of the work.

MOF‐74 (Cu) nanofibers provide a compelling alternative material for this application, enabling the formation of a rigid, yet resilient, bulk material that can withstand the operational demands of low‐pressure air bearings. Moreover, the direct casting of MOF fibers into the required geometry significantly simplifies the manufacturing process, reducing costs and avoiding the need for high‐precision machinery. By offering a scalable, cost‐effective method for producing air‐bearing pucks, MOF‐74 (Cu) nanofibers have the potential to revolutionize this field, addressing the limitations of current materials while maintaining high performance. In this work, we detail the synthesis of MOF‐74 (Cu) nanofibers, the fabrication of interwoven film and bulk materials, and their application in air‐bearing systems, widening the viability of MOF materials in real‐world applications (**Figure** **1**).

## 2 Results and Discussion

X‐ray powder diffraction confirmed the structural integrity of the synthesized MOF‐74 (Cu) nanofibers, which exhibit high aspect ratios, with diameters ranging from 20 to 100 nm and lengths from 3 to 20 µm.^[^ ^23^, ^24^ ^]^ This, combined with their flexibility in suspension, enables the formation of free‐standing films with controllable thickness and 3D structures. These mat‐like materials generally undergo brittle failure under mechanical stress but, when reinforced with other materials, form durable, interwoven composites that can be precisely shaped by drop‐casting onto desired surface geometries. The resulting structures possess sufficient strength to withstand moderate air pressure, allowing them to function as aerostatic bearings.

### 2.1 Synthesis of MOF‐74 (Cu) Nanofibers

**Figure 2:**
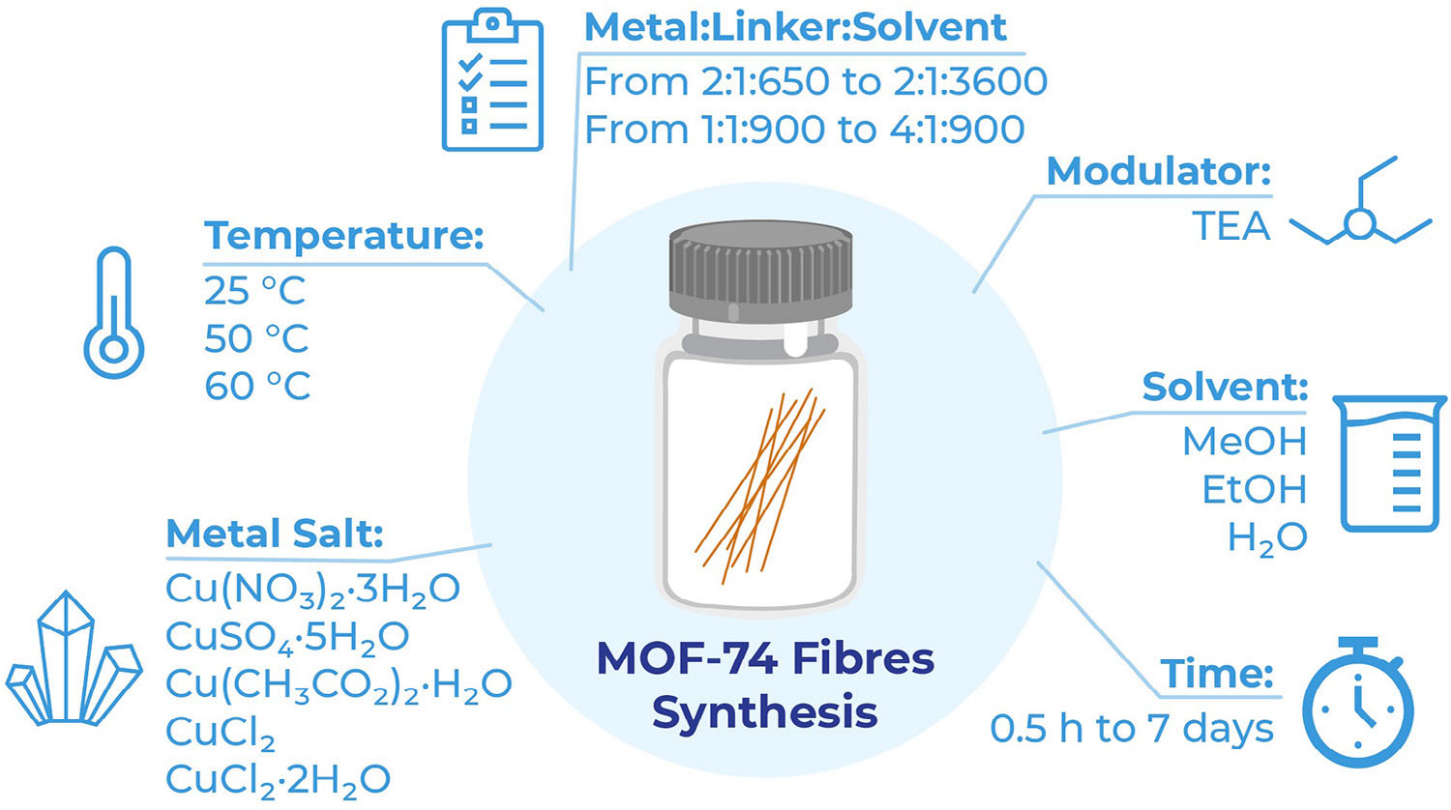
The parameters studied in the synthesis of MOF‐74 (Cu) fibers.

The synthesis of MOF‐74 (Cu) nanofibers proved robust, consistently yielding fibers across a broad range of stoichiometries and concentrations. The synthetic conditions can be considered environmentally friendly, as the synthesis is performed at room temperature in a benign solvent (methanol).^[^ ^25^, ^26^ ^]^ Fibers form rapidly, but extended reaction times notably enhance the yield, especially with the addition of a weak base as a modulator, which does not impact particle morphology. To establish the robustness of nanofiber formation across a wide range of reaction conditions, multiple synthetic parameters were studied. The impact of reagent concentration, stoichiometry, temperature, metal precursor, solvent choice, modulation and time were systematically investigated (**Figure** **2**).

#### 2.1.1 Time‐Resolved Particle Growth

**Figure 3:**
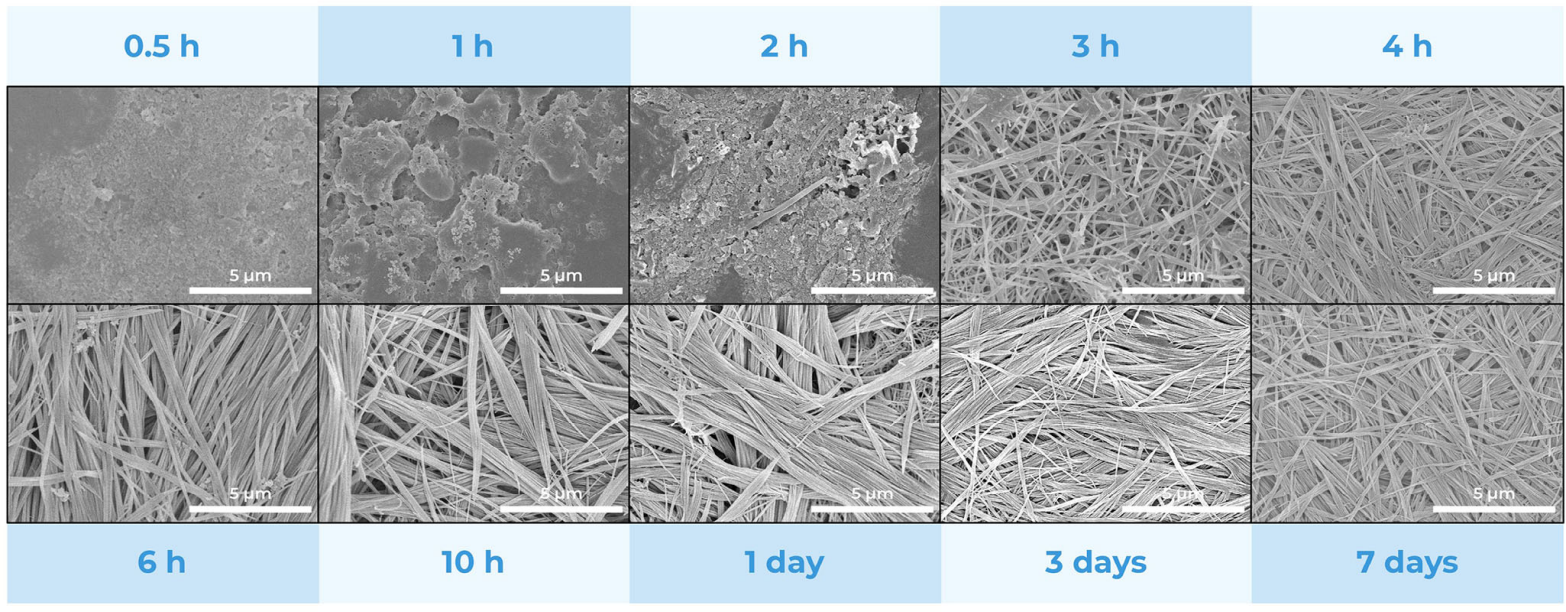
SEM images of the time‐dependent growth of MOF‐74 (Cu) nanofibers from 0.5 h to 7 days.

The growth of MOF‐74 (Cu) nanofibers was studied by collecting samples at various time points: 30 min, 1 h, 2 h, 3 h, 4 h, 6 h, 10 h, 1 day, 3 days, and 7 days (**Figure** **3**). In the first stage of the reaction, the particles appear small and with undefined morphology. The first fiber‐shaped particles appear after 2 h, although fibrous structures remain sparse, with a prevalence of the smaller, amorphous particles. After 3 h, the situation radically changes, with almost only fibers present in suspension. After 6 h, no small particles are left anymore, and the well‐defined elongated morphology remains unaltered for the remainder of the synthesis, with the clear formation of larger bundles of fibers. The PXRD patterns in Figure S1 (Supporting Information) support these observations, showing the increase of crystallinity over time. After 0.5 h of particle growth, the pattern displays broad, low‐intensity peaks, indicating low crystallinity. After 1 day, the PXRD diffraction pattern aligns closely with the simulated profile for MOF‐74 (Cu), with sharp and intense peaks, confirming the formation of highly crystalline nanofibers. This increase in crystallinity correlates well with SEM images, indicating that prolonged reaction time is crucial for achieving fully formed, highly crystalline MOF‐74 (Cu) nanofibers. The yield of the reaction is, of course, also affected by reaction time, with a maximum of 21% being reached after 3 days. As further aging of the fibers did not lead to a significant increase in neither particle crystallinity nor yield or enhanced aspect ratios, we stopped the reaction after 3 days for the rest of the presented experiments.

#### 2.1.2 Reagent Concentration and Stoichiometry

Regarding reagent concentrations, we focused on a broad range of dilutions, spanning from 2:1:650 to 2:1:3600 (metal:linker:solvent), with the higher limit dictated by the solubility of the metal salt and the lower as the last ratio at which particles are formed. All the concentrations tested yielded consistent fiber morphology, confirming the flexibility of the process (Figure S2, Supporting Information). A ratio of 2:1:900 was selected for routine synthesis, as this minimized the risk of unreacted metal salt remaining after the reaction while conserving solvent and maximizing product yield. Variations in metal‐to‐linker stoichiometry (1:1, 2:1, and 4:1) did not significantly alter fiber length or morphology, indicating that the process is highly tolerant also to stoichiometric fluctuations (Figure S3, Supporting Information). It is worth noticing that ratios of metal‐to‐linker higher than 1:1 did not afford any precipitate. For the remainder experiments we focused on a ratio of 2:1, as it is the material stoichiometric ratio, thus avoiding wasting reagents during synthesis.

#### 2.1.3 Temperature

Temperature was found to be an important factor in controlling particle morphology. Reactions at elevated temperatures (50 and 60 °C) resulted in fiber formation, but SEM analysis revealed an increased number of fragmented particles, particularly at 60 °C. This suggests that higher temperatures may induce thermal stress, leading to fiber breakage during growth, as it can be seen in Figure S4 (Supporting Information). Since the yield did not improve significantly with temperature, room temperature was identified as the most effective condition for achieving intact nanofibers.

#### 2.1.4 Metal Precursor

We tested five different copper salts for the material synthesis: Cu(NO₃)₂, CuSO₄, Cu(CH₃CO₂)₂ in their hydrate forms and CuCl₂ both as hydrate and anhydrous salts. The choice of metal precursor influenced quite strongly the success of fiber formation. While both Cu(NO₃)₂·3H₂O and CuSO₄·5H₂O led to fiber formation, both forms of CuCl_2_ failed to form any precipitate. Cu(CH₃CO₂)₂·H₂O, displayed a peculiar behavior: the direct synthesis let to the formation of a green solid with lamellar morphology and a crystalline structure incompatible with MOF‐74 (Cu). However, after carefully washing the green solid, its aging in methanol lead to the formation of MOF‐74 (Cu) fibers. As evidenced by Figure S5 (Supporting Information), the morphology of the material gradually changes over the span of a few days, from a mixture of lamellar structures and fibers at day 1 to the complete conversion into fibers at day 7. The most plausible cause for this behavior is the presence of acetate ions during the synthesis, that would modulate the reaction toward the synthesis of a structure in which the organic linker coordinates the metal nodes by only interacting via its carboxylic groups, and not with both carboxylic and phenolic groups as in the MOF‐74 structure. The removal of the acetate ions would allow the synthetized structure to slowly convert in the more stable chain‐like structure of MOF‐74.

Amongst the successful salts, Cu(NO₃)₂ was preferred due to its higher solubility in the solvent of choice. It is important to notice that all salts tested led to a small but noticeable amount of basic copper carbonate contamination, clearly visible via SEM imaging as the presence of small globular particles, usually agglomerated in larger clusters, dispersed throughout the fibers (Figure S6, Supporting Information). We found that both post‐synthetic washing of the particles with DMF or filtering the copper nitrate solution twice through 0.22 µm filters just before synthesis effectively removed the carbonate contamination. We preferred the second methodology for the experiments presented in this work, as it provides a much more environmentally benign alternative to DMF washing, although the occasional globular particle may still be present in SEM images.

#### 2.1.5 Solvent

Solvent choice revealed to be one of the most critical variables in synthesis optimization, as methanol is the only solvent in which MOF‐74 (Cu) nanofibers form, amongst the ones tested. In order to keep the synthesis green and ensure decent solubility of the reagents while still changing significantly the solvent properties we determined water and ethanol to be viable options. The synthesis carried out in ethanol showed a similar behavior to the one performed in methanol with copper acetate: the material formed during synthesis was not MOF‐74 (Cu), but a different material. Again, by aging the obtained solid in methanol, the lamellar structure evolved in MOF‐74 (Cu) nanofibers. Water led again to similar results, with the synthesis of a different crystalline material, that again could transition in MOF74 (Cu) nanofibers over the course of a few days when dispersed in methanol. Overall, the three materials obtained by using copper acetate as precursor or conducting the reaction in water or ethanol shared precise characteristics: they were insoluble in methanol (contrary to the reaction precursors) and all afforded MOF74 structures when soaked in methanol for a few days. Although the X‐ray diffraction patterns of the materials do not coincide, nor their crystallinity is sufficiently good to allow for precise structure determination (see Figure S7, Supporting Information), these characteristics led us to hypothesize that all are reticulated materials that in methanol, over time, undergo a phase transition to the thermodynamically more stable MOF‐74 structure.

#### 2.1.6 Modulation

In order to improve the reaction yield, we tested a basic modulator to increase linker deprotonation, thus facilitating faster and more complete coordination of the metal centres. In order to avoid pH related alteration of the material structure, as MOF‐74 (Cu) is sensitive to both acidic and basic conditions, we choose TEA. This tertiary amine is cheap, relatively environmentally friendly and proved very effective in quickening the synthesis of other pH sensitive MOFs, such as ZIF‐8 and ZIF‐67.^[^ ^27^ ^]^ At a TEA‐to‐linker ratio of 1:10, the yield doubled after one day (from 10% to 23%) and significantly improved after three days (from 21% to 33%) without altering the fiber morphology, as can be seen in Figure S8 (Supporting Information).

#### 2.1.7 Synthesis Scale‐Up

The synthesis was successfully scaled up by a factor of 10, from 20 mL to 200 mL, without any significant change in fiber morphology or yield. In addition, an alternative method combining solid reagents directly in the solvent, rather than dissolving them separately, yielded comparable fibers (Figure S9, Supporting Information). However, this method did not allow the removal of Cu(CO₃) from the starting metal solution, so it was only employed with freshly opened high‐purity copper nitrate.

#### 2.1.8 Particle Length Modulation

The elongated fibrous morphology, while ideal for creating films and webs, presents challenges in its direct use as a colloidal suspension due to rapid settling, and the material presents slower guest adsorption and desorption kinetics, due to less accessible openings of the 1D pores. To address these limitations, we explored the use of ultrasonication to reduce fiber length, enhancing their suspension stability and quickening adsorption kinetics, with a particular focus on the role of suspension media. In this direction, we tested fibers suspensions with a concentration of 2.3 mg mL^−1^, dispersed in methanol, acetone, DCM and toluene, in order to screen a good range of different viscosities, densities and solvent polarities. After 2 h of sonication at constant power and temperature, the different solvents proved to have a strong effect on the sonication outcome. In particular, acetone and toluene led to the shortest particles, with 0.69 and 0.87 µm of average length, respectively. Methanol led to quite longer particles, with an average length of 1.2 µm. The distribution of particle lengths follows the same trend, with a standard deviation of 0.34 for acetone, 0.40 for toluene and 0.48 for methanol. Surprisingly, DCM led to almost no change in particle length after 2 h of sonication with particles over 10 µm in length and minimal breakage visible (Figure S10, Supporting Information). These results suggest that solvent density is the most important factor, in combination with solvent viscosity, while polarity and sound speed in the medium seem not to play a major role in length modulation of the fibers.

### 2.2 Free‐Standing MOF Webs

The processability of MOF‐74 (Cu) fibers offers remarkable flexibility in shaping and fabricating free‐standing structures. In this section, we explore the formation of fiber‐based webs and films under different experimental conditions. By examining the effects of dispersion media, we evaluate how the organization of fibers influences the final material properties. Furthermore, two distinct techniques for creating thin sheets are compared, highlighting the ability to modulate sheet thickness and shape for potential applications. The integration of fibers into complex geometries and the repairability of the resulting films are demonstrated, providing insights into the mechanical robustness and versatility of these webs. Additionally, we investigate the potential for composite formation by incorporating external reinforcing materials to improve the mechanical properties of the webs. The results presented here provide a foundation for further development of MOF‐based materials for advanced applications in sensing, device fabrication, and functional coatings.

#### 2.2.1 Effect of Dispersion Media on Fiber Organization

In order to determine the best dispersion media for particle deposition, we tested six different solvents, within a wide range of solvent polarity: N,N‐dimethylformamide (DMF), methanol (MeOH), acetone (Ace), dichloromethane (DCM), toluene (Tol) and hexane (Hex). In order to exchange the solvents, we decanted the particles for two hours, removed the supernatant and exchange it with the desired solvent, repeating this process three times. As hexane is not miscible with methanol, this particular solvent exchange was mediated by and intermediate step, exchanging methanol with toluene and then toluene with hexane. It is immediately clear that solvent polarity plays an important role in the colloidal stability of the fibers. As shown in Video S1 (Supporting Information), the particles settle more slowly the higher the solvent polarity. In toluene and, even more strongly, hexane, the most apolar solvents tested, the particles actually flocculate while in suspension, aggregating before settling to the bottom of the vial. This behavior has an interesting development: as the particles settle, the flocculate sustains the particle weight, thus forcing the final height of the deposit to remain much higher than in the other solvents (Figure S11, Supporting Information). This different particle sedimentation leads to the formation of films with higher interstitial voids between fibers bundles, a useful property for the development of aerostatic bearings. As toluene provided fast settling times, a good open time for particles to spread and settle before evaporating without taking too long to dry, and, contrary to hexane, needed only one solvent exchange from methanol, thus we choose this solvent as the standard for the experiments presented in this work.

#### 2.2.2 Thin‐Sheet Fabrication

**Figure 4:**
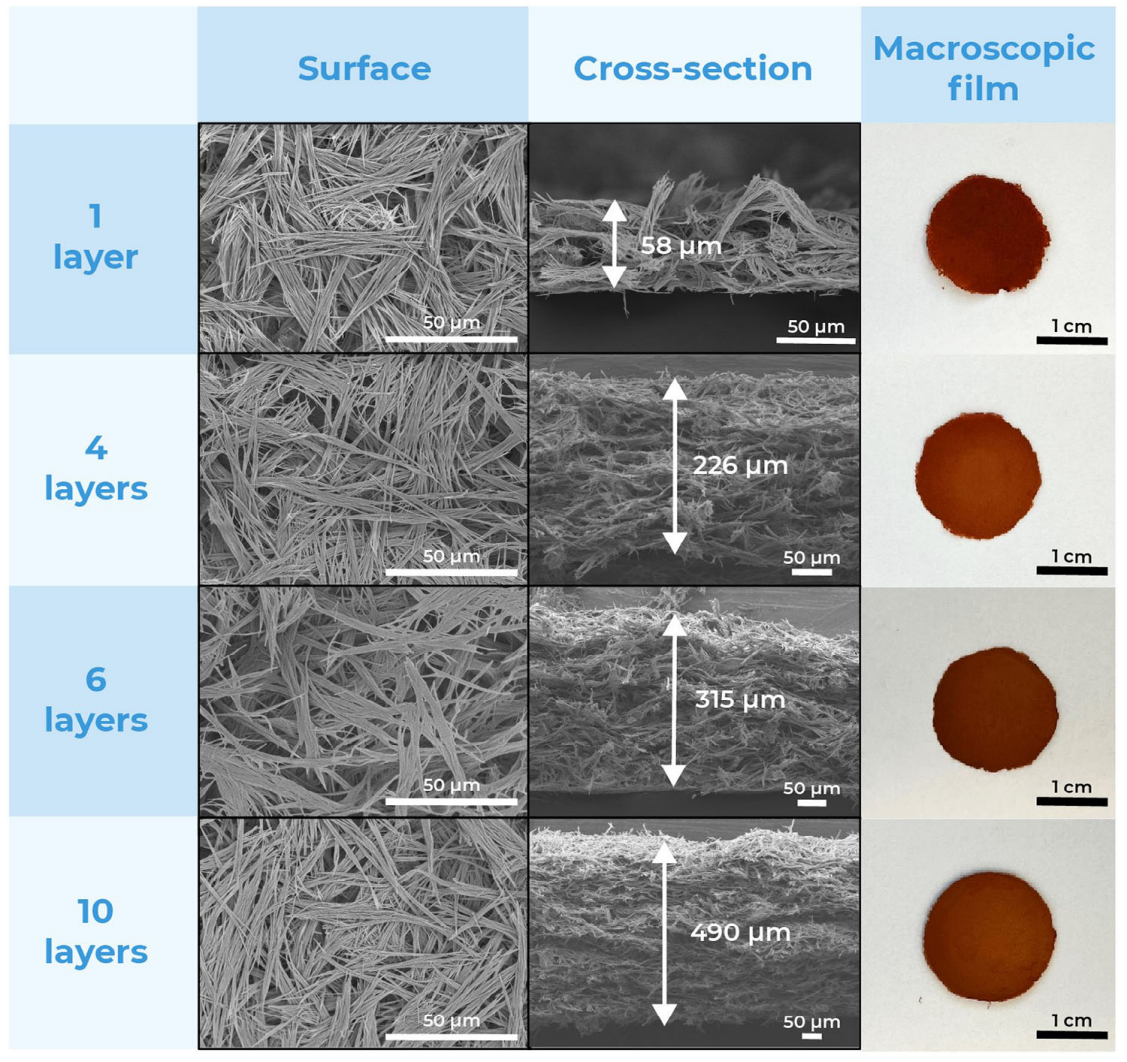
SEM and photo images of MOF‐74 (Cu) webs, casted with a different number of layers.

In order to fabricate thin sheets of fiber webs, we used two different techniques. First, we simply deposited an even layer of concentrated fibers suspension on a flat support, letting it dry slowly in air. This methodology resulted in strong films that could be easily peeled off the support when using a suitable substrate, creating films and sheets of variable and tuneable thickness. Higher thickness could be achieved by adding more aliquots of the suspension before the complete drying of the previous layer, which ensured good entanglement of the fibers. **Figure** **4** shows the surface and cross‐section of sheets created with 1, 4, 6, and 10 layers of a 100 mg mL^−1^ suspension, using 250 µL per layer, over a round surface with a diameter of 2 cm. As can be seen in Figure S12 (Supporting Information), particle casting does not affect material crystallinity, and X‐ray fluorescence confirms the presence of copper in the material structure, without traces of other metals. In our experience, the only support that could reliably and consistently release the fibers has been PTFE; the MOF strongly adheres to glass, stainless steel and other polymeric substrates, in such a way that removal attempts break the film before releasing it from the substrate. Although inconvenient for the creation of free‐standing sheets and objects, the strong adhesion of the fibers to the substrates may be a reliable way to fix it in place in the design of sensors and devices without the need for glues or other fixing agents. These may compromise or interfere with the adsorptive properties of the material particularly by clogging the material pores.

#### 2.2.3 Shaping of Thin‐Films

**Figure 5:**
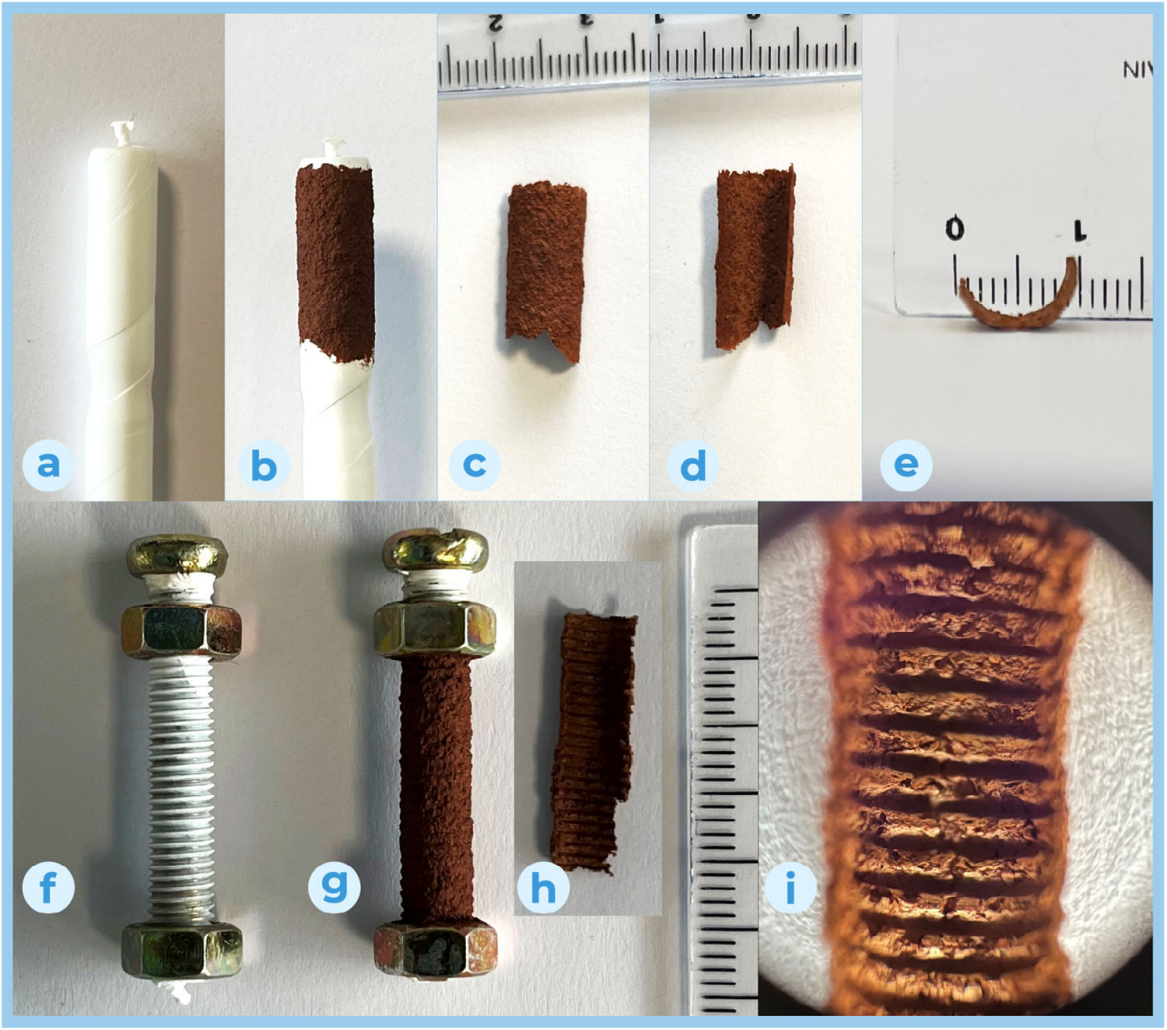
Cylindrical sheet forming: a) mandrel; b) mandrel with attached film; c) free standing shaped film, external surface; d) free standing shaped film, internal surface; e) free standing shaped film, cross section. Sheet forming over an M5×0.8 bolt template: f) mandrel; g) mandrel with attached film; h) free standing shaped film, internal surface; i) optical 10x zoom of internal surface.

The methodology described above afforded large flat sheets but proved to be impractical for more complex shapes. This is because the mould should be milled or formed directly out of solid PTFE, which is wasteful, expensive and of limited precision. In order to overcome this limitation, we were inspired by traditional paper making, in which screen moulds are used to sieve the cellulose pulp. We used PTFE tape commonly used as a thread sealant to cover complex shapes, stretching it across the surfaces in order to separate the polymer fibers that constitute the tape. This allowed us to cover the desired shapes with a thin film of releasing material (less than 20 µm thick) that enables dry fibers to be released from the mould surface and at the same time permits solvent percolation through the tape, quickening the drying of the layer. This technique allowed us to follow tight curves, such as a 4 mm radius cylinder (see **Figure** **5**). The best strategy we developed to prevent distortions in the sheet geometry during drying is to lay a thin layer of suspended particles over the desired surface and let it dry to a state of dampness, in such a way that the addition of further layers allows the first layer to set at a defined geometry but without any visible inter‐layer seam noticeable with SEM imaging of material cross‐sections. To test the limit of the fidelity with which the fibers can replicate the morphology of an object we decided to use a M5×0.8 bolt as template. The PTFE tape was stretched over the bolt by wrapping it tightly over the threads in a spiral (as for the cylinder) and running the appropriate nut along the threads for three times. The 60° threads, 0.8 mm in pitch, were indeed replicated by the film, but not fully. In Figure 5i is clearly visible how the crests of the thread on the bolt were replicated in the web, but the valleys of the same threads appear to be missing. The fibers were indeed able to deposit here, but the high surface area, combined with the small cross‐section of the thread valleys and the high propensity of the fibers to stick to any surface, concurred in breaking the top most regions of the web during release from the mould. Overall, we were still able to demonstrate a sub‐mm accuracy, but indeed further development of the methodology is required to reach higher casting fidelity, in particular for thin and unsupported areas.

#### 2.2.4 Web Bonding and Redispersion Resistance

While investigating the best methodology to avoid distortions and artefacts of the film geometry, we discovered another interesting property of these webs: their ability to be easily repaired in case of cracking and to join different sheets when fully dried. To achieve this, the two edges need to be joined side by side and the crack covered by a fairly concentrated layer of fiber suspension extending to few millimetres on each side. Once dried, the new fibers will bond to the two sheets and held them together (Video S2, Supporting Information). In our experiments on joining different sheets, we found no additional weaknesses along the welded seams, and the obtained sheet usually cracks in a different location when stressed. This methodology is not dimensionless, and will actually increase the local thickness of the sheet in the bonded area. It is also possible to laminate sheets, by flooding the surface of one with the fiber suspension and gently placing a second sheet over top before the solvent starts to dry (Figure S13, Supporting Information). A drawback of this method is that during drying, the bonding layer may pull inconsistently on the two sheets, causing distortions. However, this methodology is very useful when one wants to quickly grow the thickness of the sheet: if one of the bonded layers is being built up from the mould surface, it will not be significantly distorted by the procedure, especially if the top layer is thinner, saving time and particles to reach the desired thickness while maintaining a good fit to the casting surface.

The propensity of MOF‐74 (Cu) fibers to create a uniform and cohesive bulk material when layered originates from their ability to entangle and bond to themselves, creating bundles and stable joints. This leads to another interesting property of these webs, i.e., their capacity to withstand redispersion after drying. In order to test this, we left sheet samples under various solvents (DMF, methanol, acetone, DCM, toluene, hexane) for several days and did not observe any sign of particle redispersion, in terms of turbidity, color change or residues left after drying the supernatant (Figure S14, Supporting Information).

#### 2.2.5 Composite Formation

**Figure 6:**
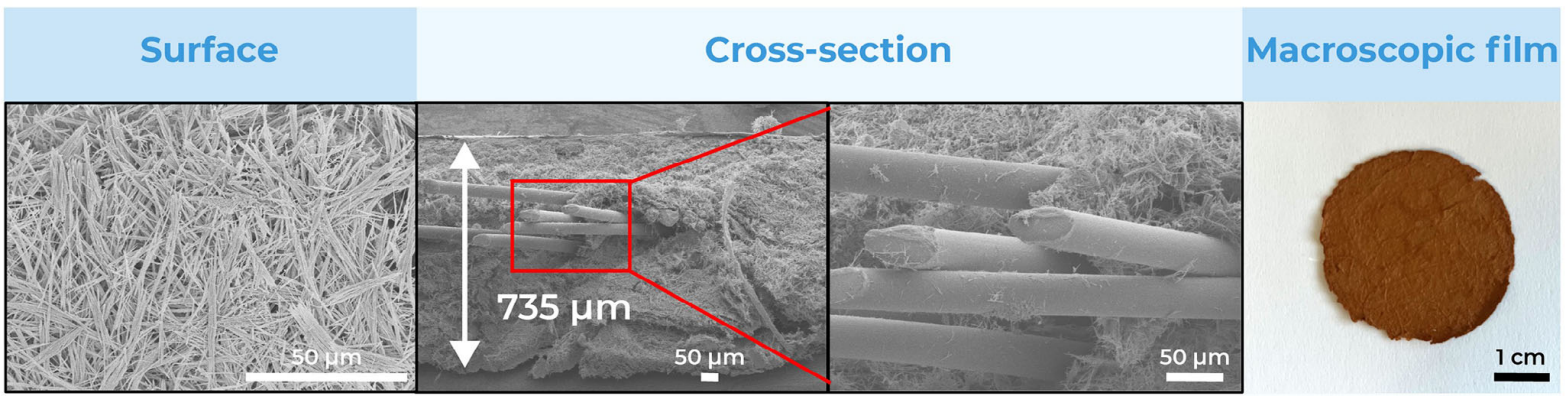
SEM images of MOF‐74(Cu) and Nylon fibers composite web.

Although the material properties of the fibers make them quite unique in terms of processability, with interesting shaping possibilities that open new design option for material integration, the resulting objects are not particularly strong or though when dry. To improve the mechanical properties of the fiber webs, we considered the formation of composite materials by integrating them with other materials in the bulk during layering. In particular, we focused on improving the resistance of the material to cracking when bent or solicited in shear. To this avail, we tested two very different materials: thin nylon strands and stainless‐steel wire, as both are cheap, chemically resistant and commonly available. We choose these materials as their combination led to the desired effect: fine nylon strands would heighten particle cohesion at a fine level, avoiding chipping and splintering of small portions of the web, thus increasing flexibility and preventing material cracking. Nylon strands proved to behave excellently in the composite, with very good dispersion and adhesion to the MOF74 fibers, as can be seen in **Figure** **6**. The stainless‐steel wire, used in its spring temper state, would stiffen the web, strengthening its structure and spreading the loads to wider web sections. The material combination led to desired outcome, with one caveat. We initially tested a stainless‐steel mesh, composed of 0.2 mm wires woven to form a 1 mm screen. However, this material proved to be not sufficiently stiff, adding little to no mechanical strength to the webs when used along the nylon fibers. Much better results were obtained with spring tempered 0.8 mm X5CrNi18‐9 (more commonly known as 304) stainless‐steel wires, which proved much more resilient to bending while still retaining good flexibility and ease of integration in the composite material.

### 2.3 Fibrous Monoliths as Castable Air‐Bearing Material

**Figure 7:**
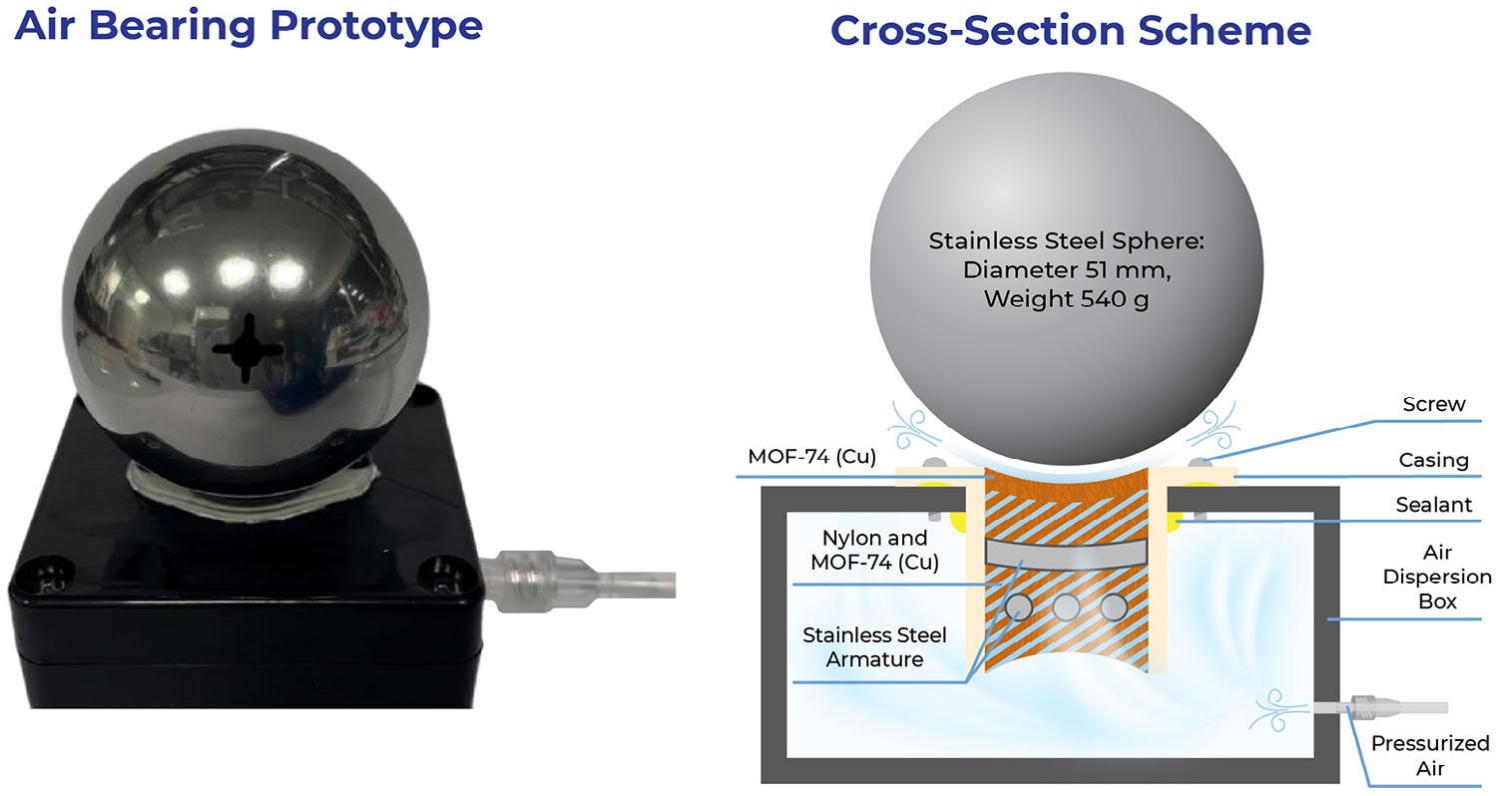
MOF air bearing prototype and cross‐section scheme of the bearing.

The bearing design process began by setting two main goals: to support a non‐flat object of substantial mass, and to achieve it using simple fabrication techniques and commonly available materials wherever possible. The chosen object for support was a stainless‐steel spherical ball bearing, 51 mm in diameter and weighing 0.54 kg, selected for its affordability and dimensional accuracy even at lower price points. Knowing the geometry and weight of the object allowed us to design the air bearing accordingly. We selected a circular aperture with a diameter of 20 mm, resulting in a bearing surface area of 3.27 cm^2^. At a pressure of 1.75 bar, this area can support roughly ten times the mass of the steel sphere, meaning only a 10% efficiency in the air bearing would be sufficient to sustain the sphere. For materials, we used disposable PE syringes to construct the restrictor scaffold, as they offer a precise and consistent diameter, and their flange easily seals against the air diffusion box, which was adapted from an ABS waterproof electrical box. The puck was cast in two phases, using a simple jig to maintain secure contact between the scaffold and the ball bearing during casting (Figure S15, Supporting Information). The primary objective of the initial casting phase was to establish the correct geometry of the bearing surface. For this, a thin layer of nanofiber was cast without any additional materials by uniformly layering 4 mL of a 100 mg mL^−1^ particle suspension in toluene over four successive layers. Each layer was added when the previous one was still damp, with most of the solvent either drained or evaporated. The second phase of casting involved additional layers of MOF‐74 and nylon fibers. During this lamination process, we also added two perpendicular layers of stainless‐steel wires, roughly shaped to match the surface curvature, to support the puck and aid in distributing the load of the compressed air. Lamination continued until a thickness of ≈8 mm was reached. The puck was then left to dry completely and was carefully detached from the master surface. The MOF air bearing prototype and the design scheme are shown in **Figure** **7**.

As demonstrated in Video S3 (Supporting Information), this additive manufacturing approach resulted in a functional air bearing, with the steel sphere lifting fully at around 1.5 bar and capable of withstanding a 14 N force before grounding at 1.75 bar. This pushes the efficiency of the fabricated air bearing over 30%. Although still below the 50% efficiency seen in commercially available graphite bearings, this result demonstrates the feasibility of additive manufacturing for air bearings and underscores the key role of MOF‐74 (Cu) nanofibers in this technological advancement. Additionally, bearing efficiency could be further improved with more sophisticated puck production techniques, such as automated casting or 3D printing, as well as by using custom‐made support components. However, the simplicity and affordability of our methodology testify to the robustness of this process.

## 3 Conclusion

This work presents a scalable, template‐free syntheti method for MOF‐74 (Cu) nanofibers, addressing a key challenge in the development of fibrous MOF morphologies. Our process produces high‐aspect‐ratio fibers that maintain their structural and chemical integrity under atmospheric conditions and exhibit flexibility for advanced material applications. These MOF‐74 (Cu) nanofibers can be processed into cohesive films, freestanding webs, and bulk materials, showcasing their potential to form stable structures with tailored geometries. Notably, we demonstrate a proof‐of‐concept application by fabricating an air‐bearing puck, which leverages the unique properties of MOF‐74 (Cu) nanofibers to support a substantial load in a contactless manner.

This approach highlights the dual benefits of MOF nanofibers: their adaptability in material processing and their performance in operational environments that demand resilience and porosity. Future improvements in manufacturing techniques, such as automated casting and advanced additive methods, could further enhance the performance and scalability of MOF‐based materials in real‐world applications.

## 4 Experimental Section

MOF‐74 (Cu) was synthesized from different copper salt solutions and 2,5‐Dihydroxyterephthalic acid (DHTP). In the typical experiment the two reagents were combined with half the required methanol for a total molar ratio of 2:1:900 (metal to linker to solvent) and stirred at 200 rpm for 30 min in order to obtain clear solutions. The two solutions were then combined in a vial or GL bottle of suitable size and the mixture was stirred at 200 rpm for 3 days. The resultant red solid was collected by centrifugation (15 000 rpm, 10 min), washed three times with methanol, dispersed, and stored under the same solvent. For modulated MOF‐74 (Cu) synthesis, triethylamine was added directly to the linker solution. Detailed conditions for each experiment can be found in the Supporting Information.

## Conflict of Interest

The authors declare no conflict of interest.

## Supporting information



Supporting Information

Supplemental Video 1

Supplemental Video 2

Supplemental Video 3

## Data Availability

The data that support the findings of this study are available from the corresponding author upon reasonable request.
