# Supplementary material for: MOF‐74 Nanofibers as an Advanced Porous Material for Air‐Bearing Technology
Source: Small. 2025 Jul 2;21(33):2411108. doi: 10.1002/smll.202411108 (PMC12372457; doi:10.1002/smll.202411108)
Supplement: Supplementary file 1 — Supporting Information [file SMLL-21-2411108-s003.docx]

**Supporting Information**

MOF-74 Nanofibers as an Advanced Porous Material for Air-Bearing Technology

Jacopo Andreo,* Xiangjiang Yu, Ekaterina Chernova, and Stefan Wuttke*

J. Andreo, X. Yu, E. Chernova, S. Wuttke

BCMaterials, Basque Center for Materials, Applications and Nanostructures, UPV/EHU Science Park, Leioa, Vizcaya, 48940, Spain

E-mail: jacopo.andreo@bcmaterials.net

S. Wuttke
Academic Centre for Materials and Nanotechnology, AGH University of Krakow, Al. Adama Mickiewicza 30, 30-059 Kraków, Poland

E-mail: swuttke@agh.edu.pl

**Table of contents**

[**1. Methodology** 3](#_Toc194052858)

[**2.1.1. Time resolved particle growth** 5](#_Toc194052859)

[**2.1.2. Reagent concentration and stoichiometry** 6](#_Toc194052860)

[**2.1.3. Temperature** 8](#_Toc194052861)

[**2.1.4. Metal precursor** 9](#_Toc194052862)

[**2.1.5. Solvent** 11](#_Toc194052863)

[**2.1.6. Modulation** 12](#_Toc194052864)

[**2.1.7. Synthesis scale-up** 13](#_Toc194052865)

[**2.1.8. Particle length modulation** 14](#_Toc194052866)

[**2.2.1. Effect of dispersion media on fibre organization** 15](#_Toc194052867)

[**2.2.2. Thin-sheet fabrication** 16](#_Toc194052868)

[**2.3. Fibrous monoliths as castable air-bearing material** 18](#_Toc194052869)

1. **Methodology**

**Materials**

Copper(II) nitrate trihydrate (Cu(NO_3_)_2_·3H_2_O, 99%, Thermo), Copper(II) sulfate pentahydrate (CuSO_4_·5H_2_O, 99%, Acros), Copper(II) acetate monohydrate (Cu(CH_3_CO_2_)_2_·H_2_O, 99%, Thermo), Copper(II) chloride (CuCl_2_, 99%, Acros), [Copper(II) chloride dihydrate](https://www.sigmaaldrich.com/ES/es/substance/copperiichloridedihydrate1704810125130) (CuCl_2_·2H_2_O, 99%, Acros), 2,5-Dihydroxyterephthalic acid (DHTP, 99.67%, BLDpharm), Triethylamine (TEA, 99%, Labkem), Methanol (MeOH, 99.8%, Labkem), Ethanol(EtOH, 99%, Labkem), Toluene (TOL, 99%, Labkem), Hexane (HEX, 99%, Sigma Aldrich), Acetone (ACE, 99%, Labkem), Dimethylformamide (DMF, 99%, Sigma Aldrich), Dichloromethane (DCM, 99%, Sigma Aldrich), Hydrochloric acid (HCI, 37%, PanReac), and Ultrapure deionized (DI) water (Adesco).

**Characterization**

**X-ray powder diffraction patterns** were collected by using a Philips X-Pert PRO automatic diffractometer operating at 40 kV and 40 mA, in theta-theta configuration, secondary monochromator with Cu*Kα* radiation (*λ* = 1.5418 Å) and a PIXcel solid state detector (active length in 2θ 3.347º). Data were collected from 5 to 75° 2θ, step size 0.026º and time per step of 700 s at RT (total time 2 h). 1º fixed soller and divergence slit giving a constant volume of sample illumination were used.

**SEM** images were recorded with a Hitachi S-4800 FEG-SEM operating at an accelerating voltage of 5 kV. Samples were previously coated with a 10 nm gold layer in an Emitech K550x ion sputter.

**Ultrasound-assisted breaking of the nanofibers to precise lengths**

Experimental procedure: MOF-74 (Cu) fibres suspension in methanol, acetone, dichloromethane or toluene (concentration of 2.3 mg/mL) was added to a glass vial (3 ml) and then sonicated with a Sonorex Super RK 103 H ultrasound bath (Bandelin electronic, GmbH&Co, Germany) at the power of 140 W and frequency of 35 kHz for time intervals of 2 hours. After each sonication cycle the suspensions were suitably diluted and deposited on silicon wafers, dried, gold coated and analysed with SEM. The SEM images were analysed quantitatively with ImageJ and more than 300 measurements for each sample were statistically sampled.

**X-ray fluorescence analysis** was performed with an Fischerscope x-ray system XDAL fluorimeter, with a voltage of 50 kV, a Ni primary filter and a measure time of 60 s.

**Synthetic procedure**

In the typical reaction, 133 mg of Cu(NO_3_)_2_·3H_2_O and 109 mg of 2,5-Dihydroxyterephthalic acid (DHTP) where dissolved in 10 ml of methanol each. Once fully dissolved, the solutions were combined under stirring (200 rpm) and the reaction mixture was left at room temperature, under constant stirring, for 3 days. The resultant red solid was collected via centrifugation, redispersed in methanol for three times, ensuring complete removal of unreacted species, and then stored in the same solvent.

The typical reaction parameters are the following:

**Metal precursor:** Cu(NO_3_)_2_·3H_2_O

**Organic Linker:** 2,5-Dihydroxyterephthalic acid (DHTP)

**Solvent:** MeOH

**Reaction time:** 3 days

**Reagents concentration (Cu:DHTP:MeOH):** 2:1:900

**Temperature:** 20º C

**Solvent volume:** 20 ml

Each variation will be clearly indicated for each experiment presented in this Supporting information.

**2.1.1. Time resolved particle growth**

**Aging time:** 0.5 h, 1 h, 2 h, 3 h, 4 h, 6 h, 10 h, 1 day, 3 days and 7 days





**Figure S1**: Powder X-ray diffraction patterns of MOF-74 (Cu) fibres synthetized in batch at various aging time.

**2.1.2. Reagent concentration and stoichiometry**

**Reagents concentration (Cu:DHTP:MeOH):** X:1:X


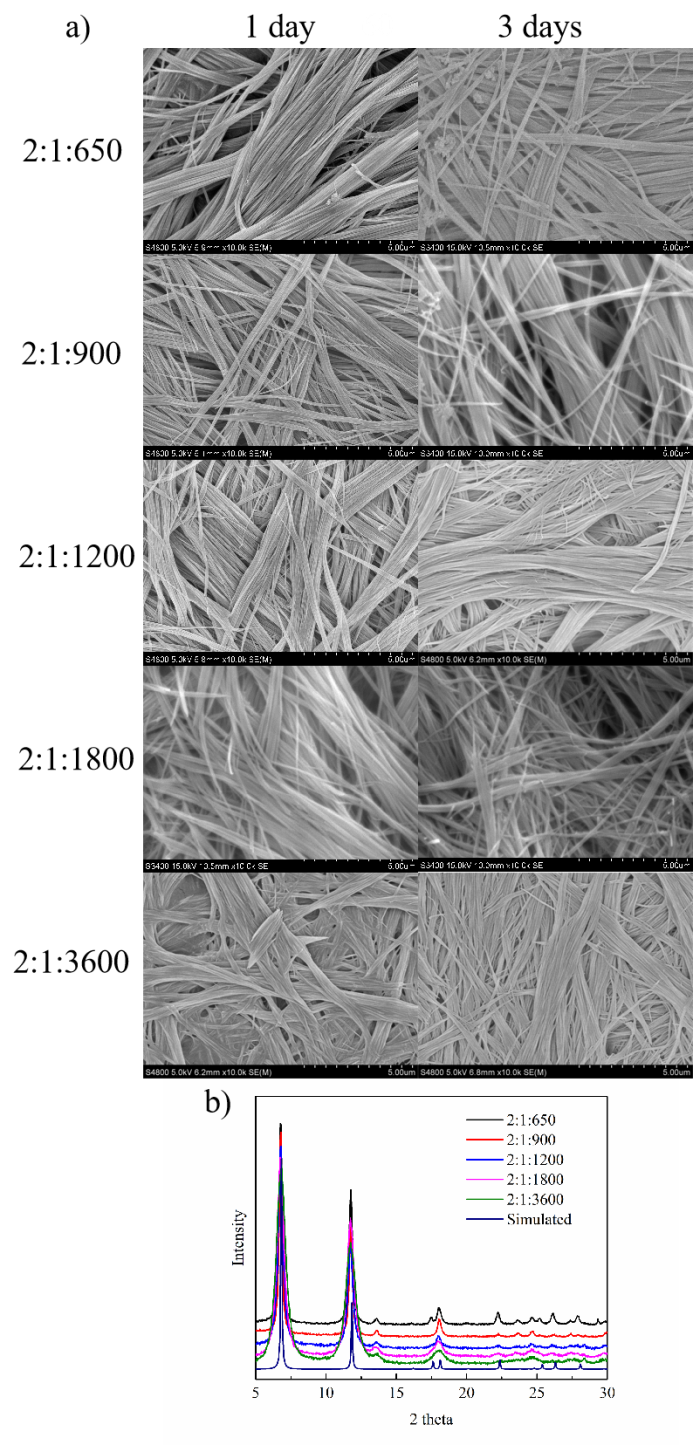


**Figure S2**: a) SEM images and b) powder X-ray diffraction patterns of MOF-74 (Cu) fibres synthetized in batch at different concentrations with 2:1 reagent ratio, after 1 day of reaction.


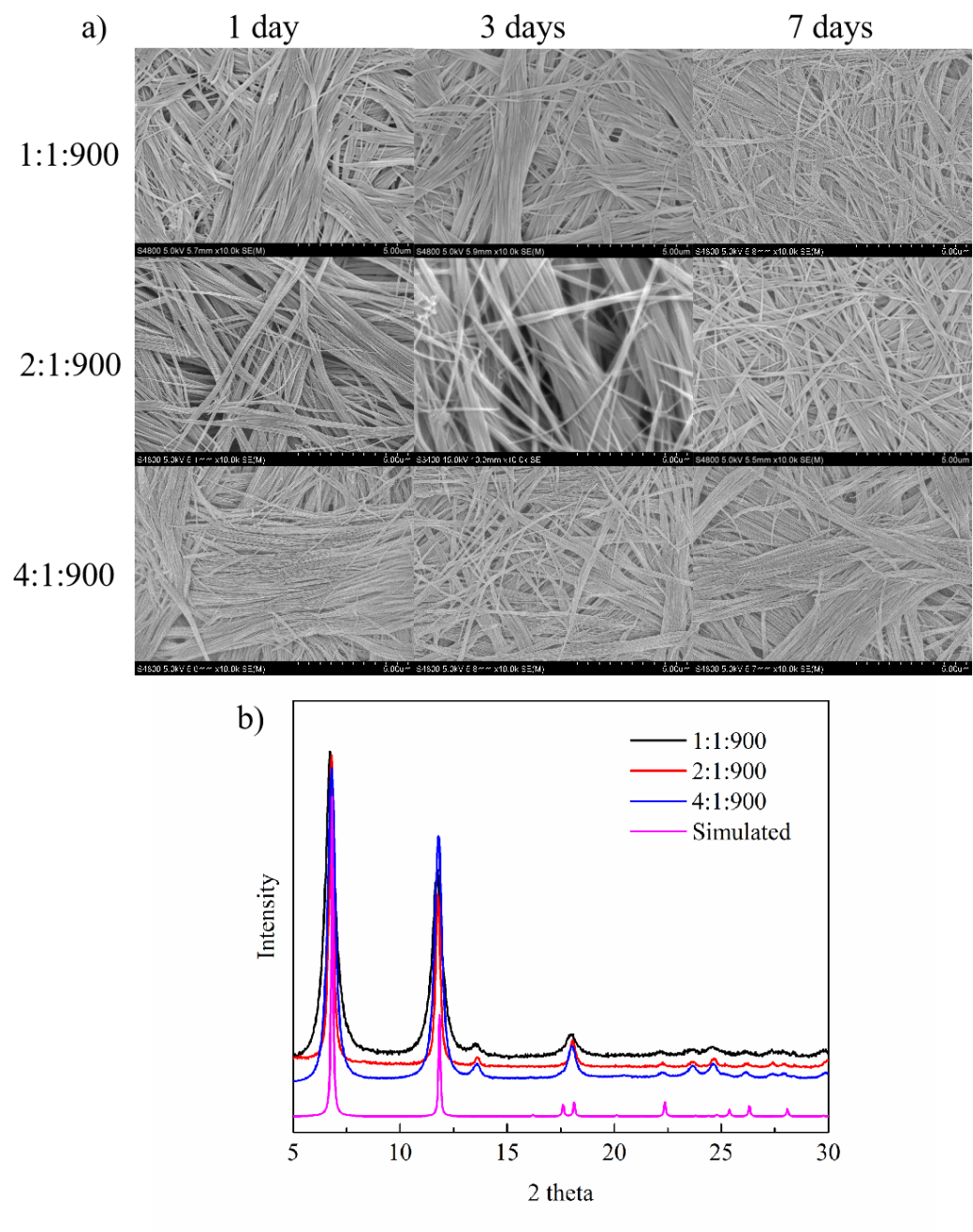


**Figure S3**: a) SEM images and b) powder X-ray diffraction patterns of MOF-74 (Cu) fibres synthetized in batch at different reagents stoichiometries, after 3 days of reaction.

**2.1.3. Temperature**

**Temperature:** 25, 50 and 60 ºC


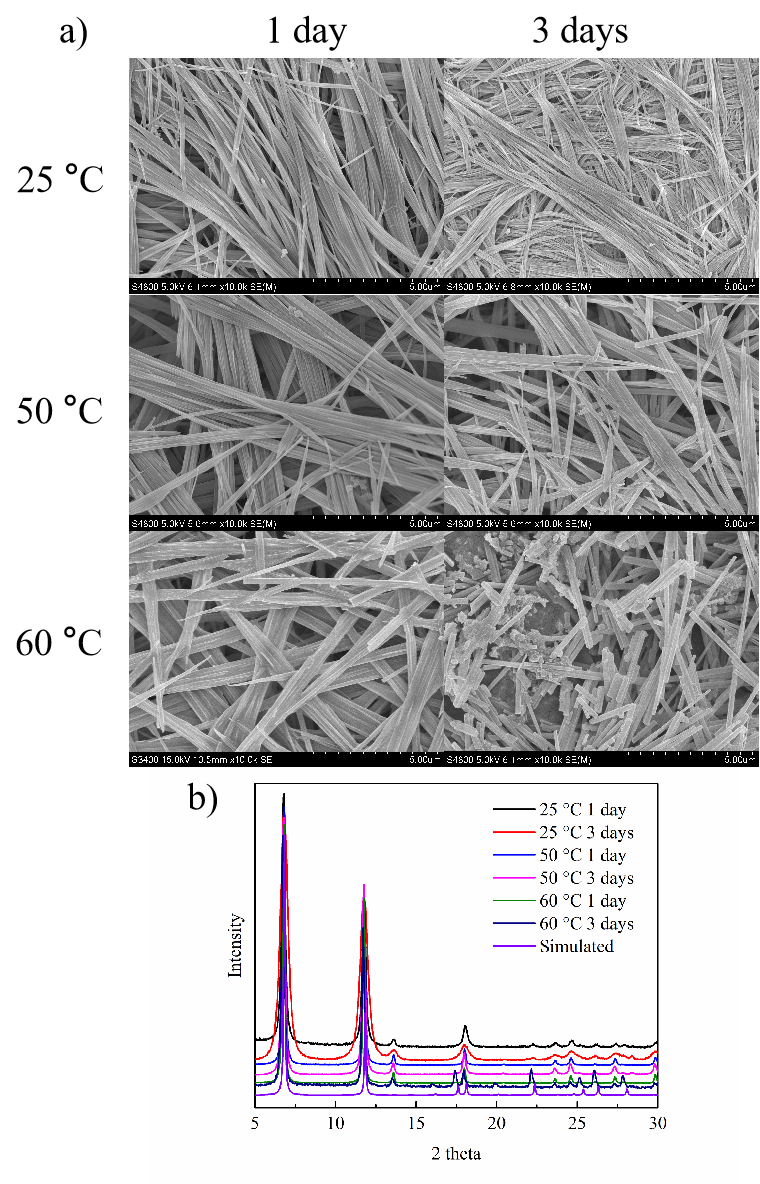


**Figure S4**: a) SEM images and b) powder X-ray diffraction patterns of MOF-74 (Cu) fibres synthetized in batch at different temperatures.

**2.1.4. Metal precursor**

**Metal precursor:** Cu(NO_3_)_2_·3H_2_O, Cu(CH_3_CO_2_)_2_·H_2_O, CuSO_4_·5H_2_O, CuCl_2_·2H_2_O and CuCl_2_.

**Aging time:** 1 day, 3 days and 7 days.


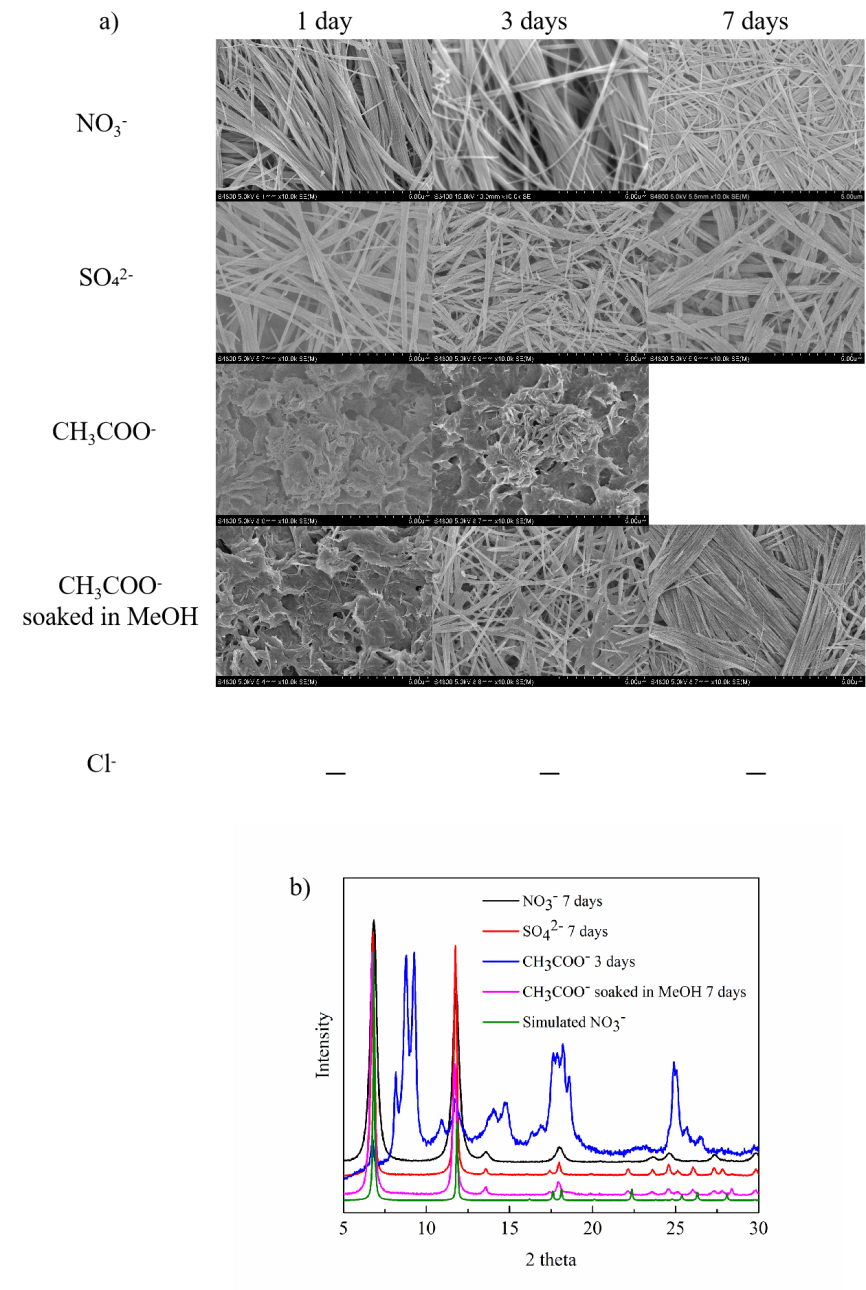


**Figure S5**: a) SEM images and b) powder X-ray diffraction patterns of MOF-74(Cu) fibres synthetized in batch at different metal source with 2:1:900 reagent ratio. CuCl_2_·2H_2_O and CuCl_2_ did not form any precipitate.


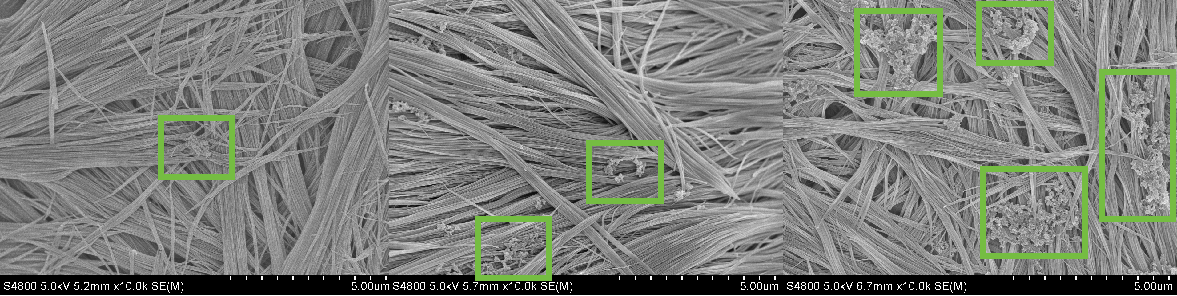


**Figure S6**: SEM images of MOF-74 (Cu) fibres synthetized in batch at different metal source with 2:1:900 reagent ratio. Basic copper carbonate particles are observed in the green box. Aging time: 3 days.

**2.1.5. Solvent**

**Solvent:** methanol (MeOH), ethanol (EtOH) and water (H_2_O)


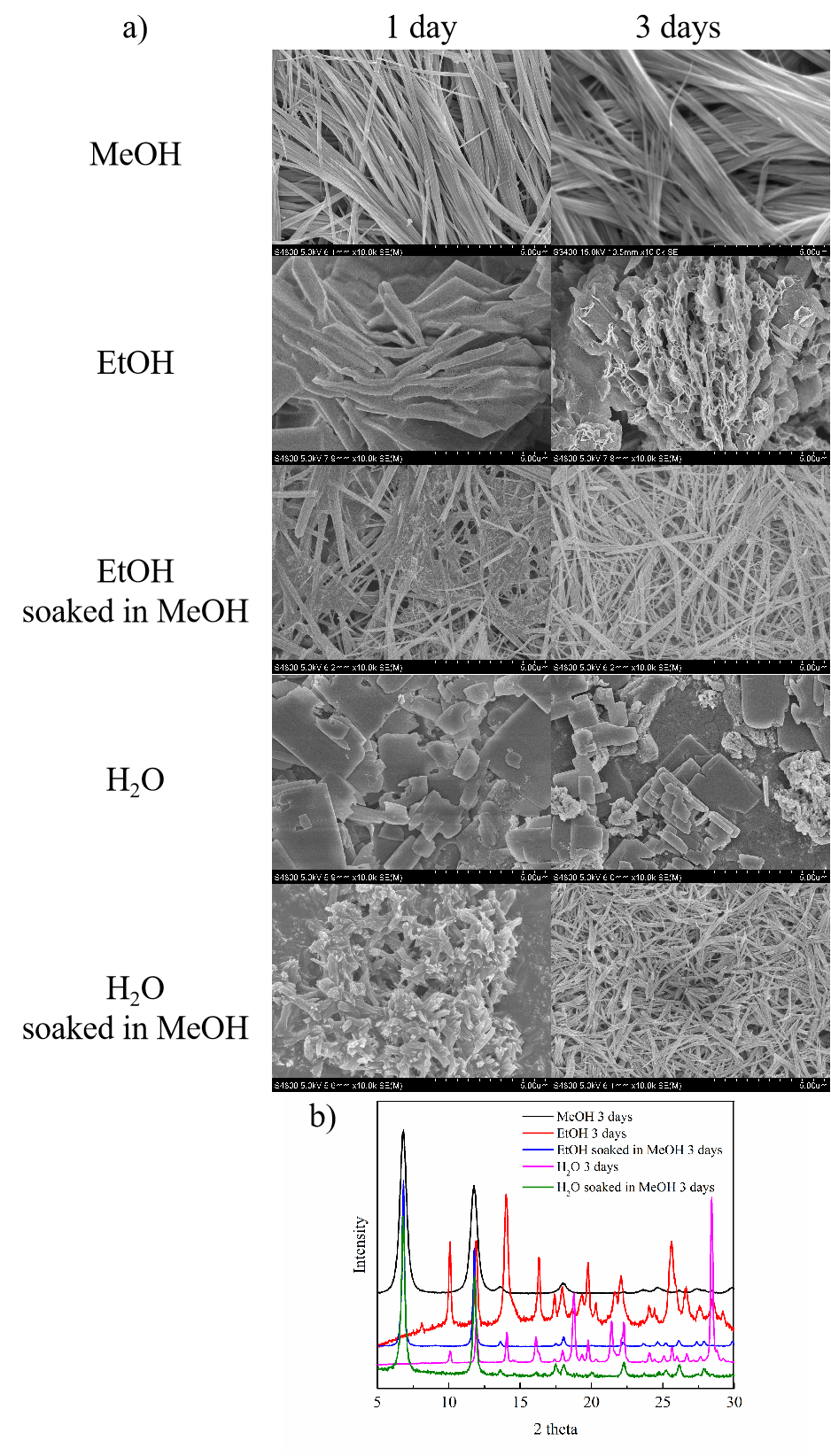


**Figure S7**: a) SEM images and b) powder X-ray diffraction patterns of MOF-74 (Cu) fibres synthetized in batch at different solvents.

**2.1.6. Modulation**

**Reagents concentration (Cu:DHTP:MeOH:TEA(Triethylamine)):** 2:1:900:0.1.

**Solvent volume:** 200 ml.


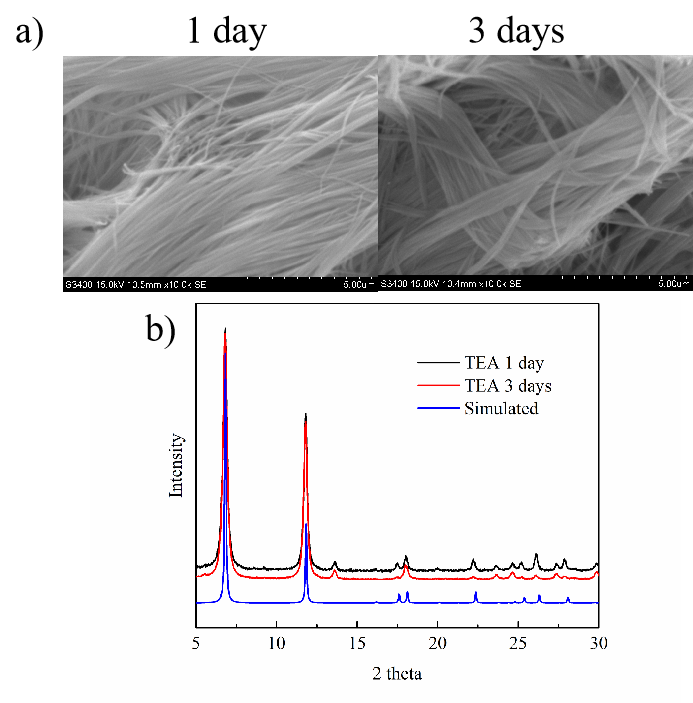


**Figure S8**: a) SEM images and b) powder X-ray diffraction patterns of MOF-74 (Cu) fibres synthetized with TEA modulation.

**2.1.7. Synthesis scale-up**

**Solvent volume:** 20 ml and 200 ml.

**1 step experiment:** direct mixing of solid metal and linker in methanol, without previous dissolution.


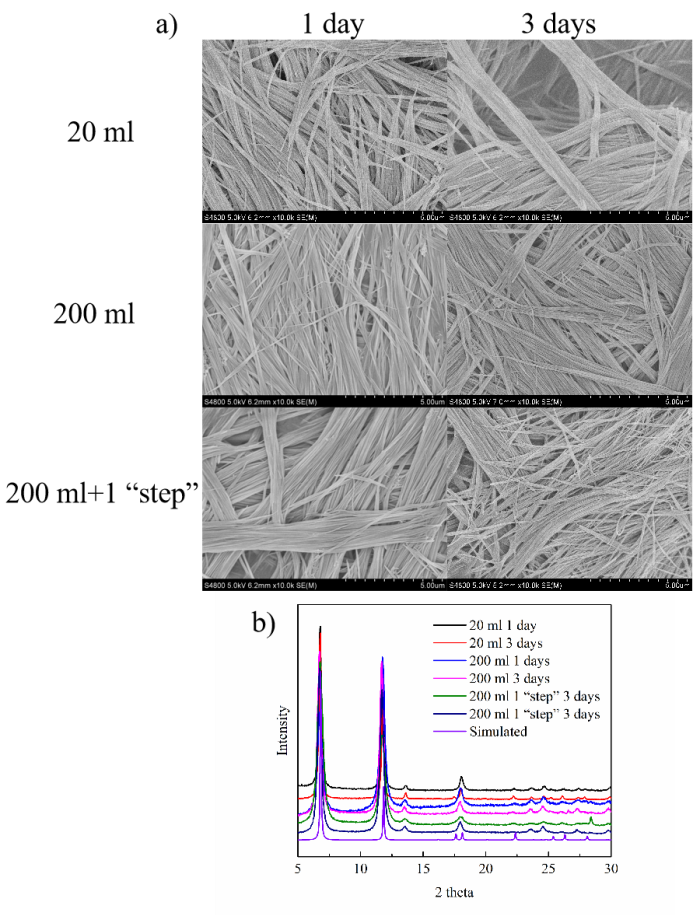


**Figure S9**: a) SEM images and b) powder X-ray diffraction patterns of MOF-74 (Cu) fibres synthetized in 200 ml Scale-up batch, in 2:1:900:0.1 reagent ratio with TEA and in different “step” experiment respectively.

**2.1.8. Particle length modulation**


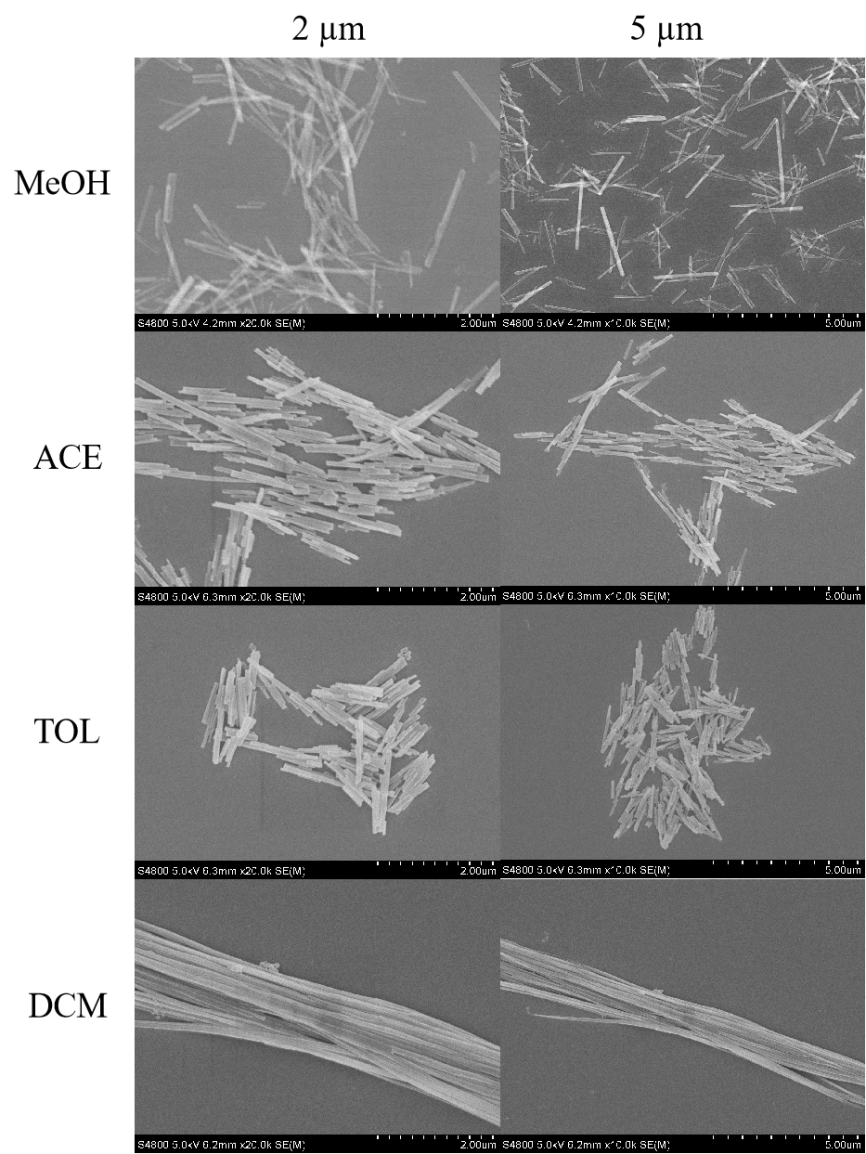


**Figure S10**: SEM images of MOF-74 (Cu) fibres after 2 h sonication in different solvents.

**2.2.1. Effect of dispersion media on fibre organization**


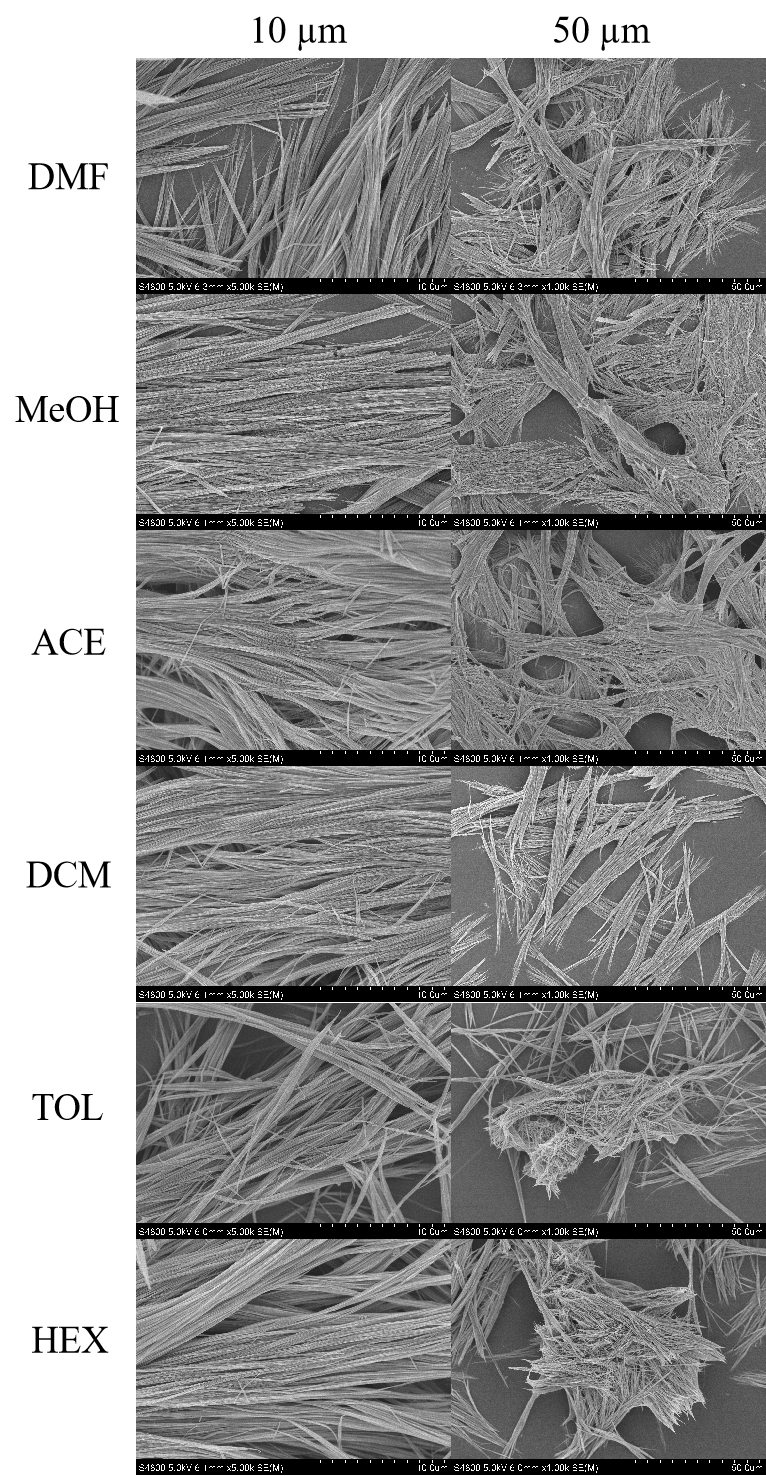


**Figure S11:** SEM images of MOF-74(Cu) fibres treated in different solvents respectively. Silicon wafer was used as the substrate.

**2.2.2. Thin-sheet fabrication**

**
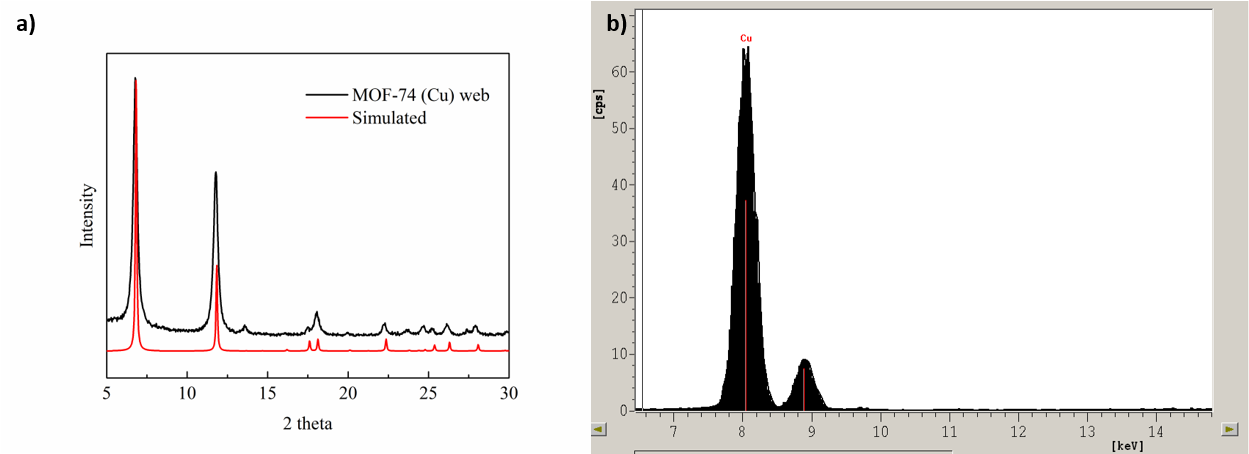
**

**Figure S12.** a) Powder X-ray diffraction pattern and b) X-ray fluorescence analysis of MOF-74 (Cu) web, casted with 4 layers.

**2.2.4. Web repair, bonding, and redispersion resistance**

**Bonding**


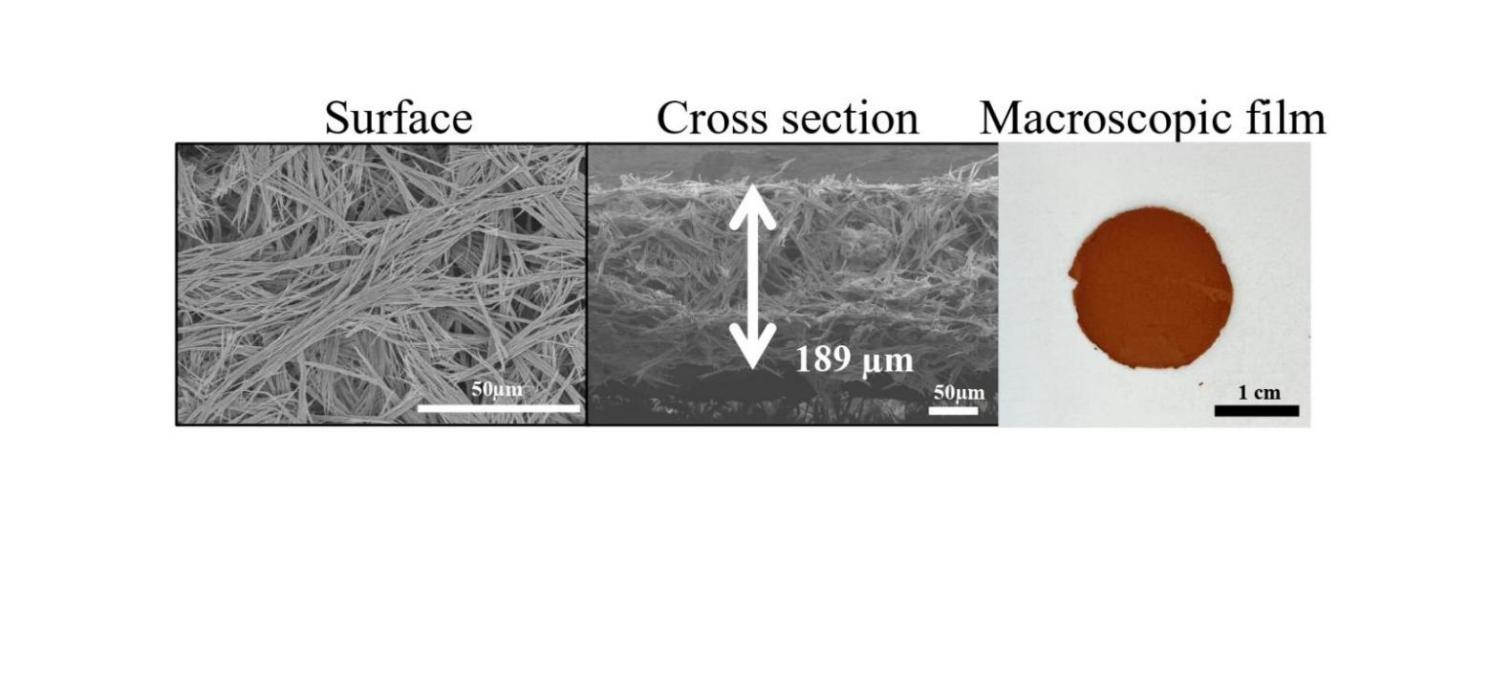


**Figure S13**: SEM images of MOF-74 (Cu) fibres laminated films. In the picture, two monolayers were casted, fully dried and then laminated with the same particle suspension (100 μl/ml, 250 μl/layer, round surface 2 cm in diameter).

**Redispersion resistance**


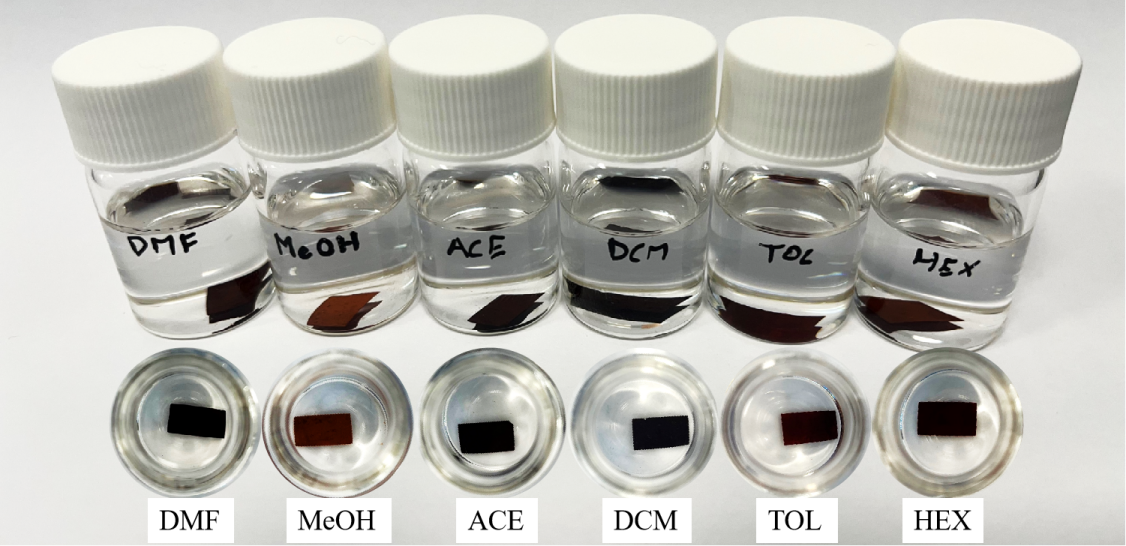


**Figure S14**: Images of the redispersion resistance of MOF-74 (Cu) webs after 7 days of soaking in different solvents.

**2.3. Fibrous monoliths as castable air-bearing material**


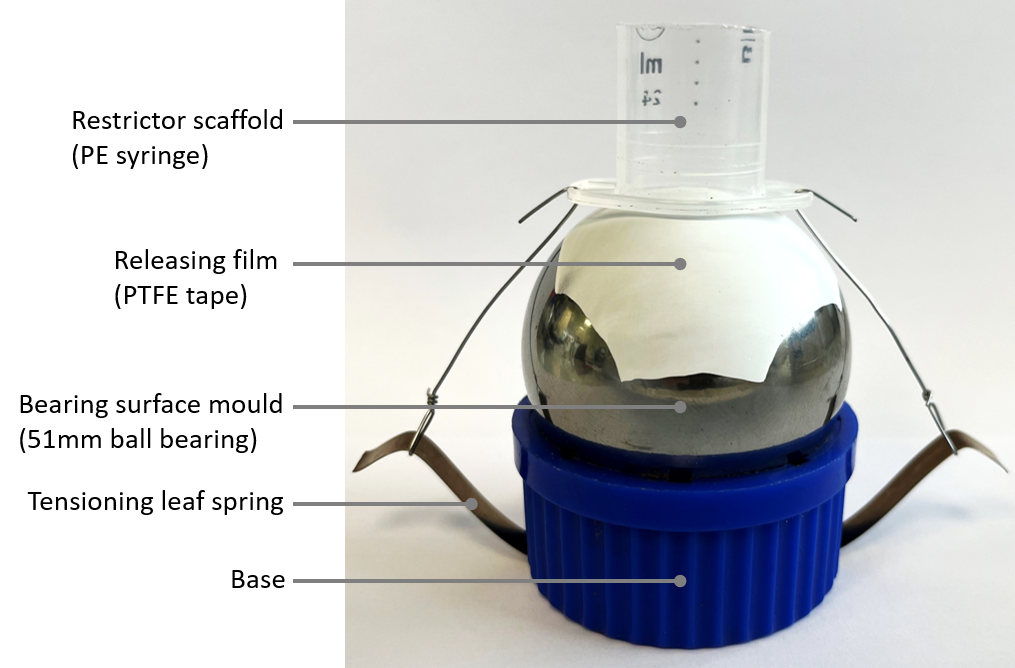


**Figure S15**: Casting jig for air-bearing pucks.
